# Inhibition of Tetraspanin Functions Impairs Human Papillomavirus and Cytomegalovirus Infections

**DOI:** 10.3390/ijms19103007

**Published:** 2018-10-02

**Authors:** Laura A. Fast, Snježana Mikuličić, Anna Fritzen, Jonas Schwickert, Fatima Boukhallouk, Daniel Hochdorfer, Christian Sinzger, Henar Suarez, Peter N. Monk, María Yáñez-Mó, Diana Lieber, Luise Florin

**Affiliations:** 1 Institute for Virology and Research Center for Immunotherapy (FZI), University Medical Center of the Johannes Gutenberg University, 55122 Mainz, Germany; laura.fast@uni-mainz.de (L.A.F.); snjezana.mikulicic@gmail.com (S.M.); fritzenaen@t-online.de (A.F.); jonas.schwickert@web.de (J.S.); 2 Institute for Medical Microbiology and Hygiene, University Medical Center of the Johannes Gutenberg University, 55122 Mainz, Germany; bouk@uni-mainz.de; 3 Institute of Virology, Ulm University Medical Center, 89081 Ulm, Germany; danielhochdorfer@gmail.com (D.H.); Christian.Sinzger@uniklinik-ulm.de (C.S.); diana_lieber@gmx.de (D.L.); 4 Departamento de Biología Molecular, UAM, 28049 Madrid, Spain; henar.suarez.montero@gmail.com (H.S.); maria.yannez@uam.es (M.Y.-M.); 5 Centro de Biología Molecular Severo Ochoa (CBM-SO), Instituto de Investigación Sanitaria Princesa (IIS-IP), 28006 Madrid, Spain; 6 Department of Infection, Immunity and Cardiovascular Disease, School of Medicine and Biomedical Science, University of Sheffield, Sheffield S10 2RX, England, UK; p.monk@sheffield.ac.uk

**Keywords:** human papillomavirus, HPV16, human cytomegalovirus, tetraspanin, virus entry, blocking peptide, IC_50_

## Abstract

Tetraspanins are suggested to regulate the composition of cell membrane components and control intracellular transport, which leaves them vulnerable to utilization by pathogens such as human papillomaviruses (HPV) and cytomegaloviruses (HCMV) to facilitate host cell entry and subsequent infection. In this study, by means of cellular depletion, the cluster of differentiation (CD) tetraspanins CD9, CD63, and CD151 were found to reduce HPV16 infection in HeLa cells by 50 to 80%. Moreover, we tested recombinant proteins or peptides of specific tetraspanin domains on their effect on the most oncogenic HPV type, HPV16, and HCMV. We found that the C-terminal tails of CD63 and CD151 significantly inhibited infections of both HPV16 and HCMV. Although CD9 was newly identified as a key cellular factor for HPV16 infection, the recombinant CD9 C-terminal peptide had no effect on infection. Based on the determined half-maximal inhibitory concentration (IC_50_), we classified CD63 and CD151 C-terminal peptides as moderate to potent inhibitors of HPV16 infection in HeLa and HaCaT cells, and in EA.hy926, HFF (human foreskin fibroblast) cells, and HEC-LTT (human endothelial cell-large T antigen and telomerase) cells for HCMV, respectively. These results indicate that HPV16 and HCMV share similar cellular requirements for their entry into host cells and reveal the necessity of the cytoplasmic CD151 and CD63 C-termini in virus infections. Furthermore, this highlights the suitability of these peptides for functional investigation of tetraspanin domains and as inhibitors of pathogen infections.

## 1. Introduction

Tetraspanin proteins play important roles during the life cycle of numerous viruses [1,2,3,4,5]. It has been suggested that virus entry involves specific tetraspanins such as cluster of differentiation (CD) tetraspanins CD9, CD63, CD81 and CD151, making them putative antiviral targets.

The widely expressed family of tetraspanin proteins is composed of 33 members in humans [6]. Tetraspanins localize in the plasma membrane and the membranes of intracellular compartments. They are structurally conserved harboring four transmembrane domains, three intracellular domains as well as a small and a large extracellular loop [7,8,9,10,11]. The large extracellular loop (LEL) of tetraspanins is a flexible functional domain with a variable sequence but different tetraspanins share a conserved structure [12,13,14]. Due to its ability to facilitate specific interactions, the LEL supports anchoring of multiple proteins to one site of the membrane [9,12]. The short cytoplasmic C-terminal tail of tetraspanins serves as a connector to cytoplasmic interaction partners [15,16,17,18,19]. Despite their relative shortness of about a dozen amino acids, some tetraspanin C-termini contain functional units, such as the C-termini of tetraspanins CD63 and CD151 that contain a tyrosine-based sorting motif essential for their subcellular localization [16,20]. Different tetraspanin domains can simultaneously interact with other tetraspanins or proteins from other protein families and thereby locally concentrate molecules in a functional network, the tetraspanin-enriched microdomains (TEMs or TERMs) [21,22]. The ability to form TEMs and co-integrate interaction partners such as growth factor receptors, adhesion molecules, and proteases into these assemblies enables local concentration of factors required for virus entry [5].

Human papillomaviruses (HPV) are small, nonenveloped DNA viruses infecting the skin and mucosa. Depending on the HPV type, infection causes either the formation of benign warts or malignant tumors. The high-risk HPV types are responsible for severe human cancers, including almost all cases of cervical cancer and numerous anogenital, head, and neck tumors [23,24]. High-risk HPV type 16 (HPV16) is most studied as it displays the highest oncogenic potential [25,26]. This HPV type requires a distinct set of receptors and accessory proteins such as tetraspanins [27,28,29], laminin-binding integrins [28,30,31,32], and growth factor receptors (GFRs) [33], for its uptake into the cell (reviewed in [34,35,36]). Tetraspanin CD151 is able to interact with these HPV16 entry factors [9,37] and mediates virus uptake via a clathrin-, caveolin- and dynamin-independent endocytic pathway [28,38,39]. Moreover, mutational analyses uncovered CD151 domains that are critical for endocytic uptake of HPVs, like the integrin-interacting domain of the LEL or the cytoplasmic C-terminus [28,39]. Following endocytosis, virions are trafficked via endosomal structures. This post-endocytic trafficking requires tetraspanin CD63 [40]. Here again, the functionality of its C-terminus is required for HPV16 infection as it is essential for the interaction with the adaptor protein syntenin-1 [16,40]. CD63/syntenin-1 complex formation ensures HPV trafficking to multivesicular bodies. This step is vital for viral capsid disassembly, and mediates the delivery of the viral genome to the host cell nucleus [40]. Additionally, HPV infection induces clustering of CD81 on T-cell membranes leading to the formation of large cluster networks required for viral uptake [29]. Therefore, in addition to CD151 and CD63, other tetraspanins might be involved in HPV infection as it has been shown for other viruses like HCMV [3,4,5,41].

Human cytomegalovirus (HCMV), an enveloped DNA virus and member of the *herpesviridae* family, can cause life-threatening disease in immunocompromised individuals and is among the leading causes of infection-related birth defects [42,43]. At present, there is no vaccine that provides sufficient protection from HCMV infection, therefore the need to identify new antiviral drug targets remains. For successful entry into certain cell types, such as epithelial and endothelial cells, HCMV requires several cellular factors such as tetraspanin proteins CD9, CD63, CD81, and CD151 [4,41], integrins [44,45], growth factor receptors (epidermal GFR and platelet-derived GFR alpha) as well as neuropilin-2 which have been separately investigated [46,47,48].

In this study, the general requirements of the tetraspanins CD9, CD63, CD81, and CD151 in infections by the most oncogenic HPV type, HPV16, were tested as previously investigated for HCMV. Although future research is required to identify the precise role of tetraspanins CD9 and CD81, our study is the first to define them as novel players in HPV16 infection. Furthermore, we analyzed the involvement of specific tetraspanin domains, the LEL and the C-terminus, in HPV and HCMV infection by using recombinant peptides or proteins comprising these domains of CD9, CD63, CD81 and CD151 tetraspanins. We demonstrate that C-terminal peptides of CD63 and CD151 exhibit a moderate to potent inhibitory effect on HPV16 and HCMV infections. These findings suggest that the function of the C-terminus of CD151 and CD63 but not of CD9 is of particular importance for virus entry and infection. In conclusion, our results highlight the C-terminal tails of CD63 and CD151 as effective HPV16 and HCMV entry inhibitors. Moreover, we find peptides of functional tetraspanin domains as useful tools to investigate the role of tetraspanins and their domains in virus infections.

## 2. Results

### 2.1. Cluster of Differentiation (CD) Tetraspanins CD9, CD63, CD81 and CD151 Are Required for Human Papillomavirus Type 16 (HPV16) Infection.

**Figure 1:**
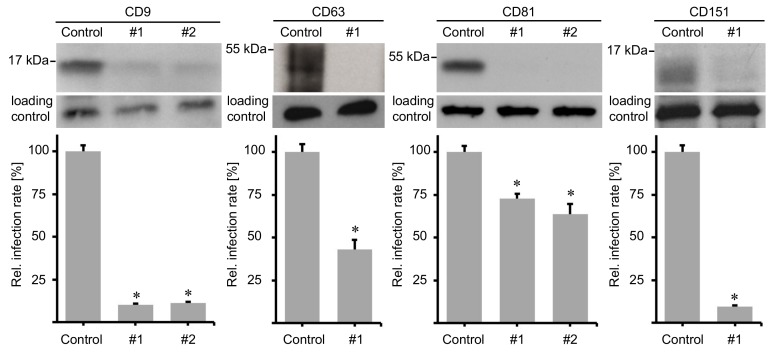
Tetraspanins CD9, CD63, CD81, and CD151 are required for HPV16 infection. HeLa cells were treated with control, tetraspanin CD9, CD63, CD81 or CD151 specific siRNAs (#1 or #2) for 48 h, then infected with HPV16 PsVs for 24 h. Upper panels show siRNA knockdown efficiency (β-actin served as loading control). Graphs presented on the lower panels show relative infection rates assessed by measuring luciferase activity since HPV16 PsVs harbor a luciferase expression plasmid. Data sets are shown as mean ± standard error of the mean (SEM) and were normalized to the mean of control siRNA treated cells (set to 100%). Asterisks indicate significant differences to the control (*p* < 0.05).

Tetraspanin assemblies play an important role in virus entry [5]. It has been shown that CD9, CD81 and CD151 were downregulated during HCMV entry and that cellular depletion of CD9, CD63 and CD151 reduced HCMV infection [4,41]. Oncogenic HPV types utilize TEMs with tetraspanin CD151 as a key player during endocytosis [28,38]. Moreover, tetraspanin CD63 is crucial for post-endocytic virus trafficking, ensuring delivery of the viral DNA to the host cell nucleus [40]. In addition, we have shown earlier that neutralizing antibodies specific for CD63 and CD151 reduced infectivity in HEK293TT cell line [27,28,29]. The importance of tetraspanins CD9 and CD81 for HPV infection has not yet been revealed. To test whether additional tetraspanins are involved in HPV16 infection, we applied a pseudovirus (PsV) infection assay, depleting either CD9 or CD81. siRNAs targeting CD151 and CD63 served as a control for impaired infection. HeLa cells were incubated with specific tetraspanin siRNAs as indicated for 48 h before infection with HPV16 PsVs. All tested tetraspanin siRNAs induced a significant reduction in HPV16 infection rates (Figure 1). Cellular depletion of CD81 induced a weak infection rate inhibition of 25 to 30%. Knockdown of CD63 displayed a reduction of infection by more than 50% and CD9 knockdown resulted in a strong inhibition of about 90%, which was comparable to results obtained by CD151 siRNAs. Since the knockdown efficiency of tetraspanin CD63 is stronger than the assessed infectivity after its depletion, it is likely that another tetraspanin might overtake the role of CD63. Together, these results emphasize the role of different tetraspanins in HPV infection and uncover the importance of CD9 for infection of HeLa cells.

### 2.2. Peptides Comprising the Large Extracellular Loop of Tetraspanins Interfere with HPV16 Infection.

**Figure 2:**
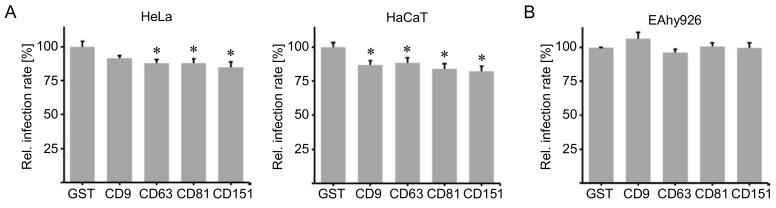
Peptides comprising the large extracellular loop of tetraspanins interfere with HPV16 infection. (**A**) HeLa and HaCaT cells were incubated with 500 nM glutathione *S*-transferase (GST) negative control protein or GST fusion proteins comprising the large extracellular loop of tetraspanin CD9, CD63, CD81 or CD151 for 3 h. Cells were infected with HPV16 PsVs and relative infection rate was assessed by measuring luciferase activity 24 h post infection (h.p.i.). (**B**) EA.hy926 cells were incubated with 500 nM GST negative control protein or GST fusion proteins of the large extracellular loop of tetraspanin CD9, CD63, CD81, or CD151 for 2 h. Cells were infected with HCMV strain TB40/E and analyzed via immunofluorescence. The fraction of infected cells was calculated by dividing the number of immediate early antigen (IE-Ag) positive cells by the number of DAPI positive nuclei 24 h.p.i. The data is shown as mean ± SEM and was normalized to the mean of control peptide (GST) treated cells (set to 100%). Asterisks indicate significant differences compared to control (*p* < 0.05).

The large extracellular loop of tetraspanin proteins is a variable functional region. It mediates tetraspanin interactions with other tetraspanin molecules or interacting partner proteins [12,13,14,29,49], thereby contributing to the formation of functional tetraspanin-enriched microdomains. Recombinant proteins comprising LELs of tetraspanins involved in HPV and HCMV entry might inhibit infection by competitively binding PsVs or tetraspanin interaction partners. To this end, LEL-GST (glutathione *S*-transferase) fusion proteins of tetraspanins CD9, CD63, CD81 or CD151 were produced and purified as previously demonstrated [50]. HeLa and HaCaT cells were incubated with these recombinant LELs for 1 h and subsequently infected with HPV16 PsVs. The LELs of CD63, CD81 and CD151 induced a minor, but significant reduction of HPV infection rates in HeLa and HaCaT cells at 24 h post infection (h.p.i.) (Figure 2A). To test whether the LELs affect HCMV infection, EA.hy926 cells were incubated with the recombinant proteins and subsequently infected with HCMV strain TB40/E. At 24 h.p.i., the cells were fixed and the fraction of infected cells expressing viral immediate early antigen (IE-Ag) was determined via immunofluorescence. None of the tested tetraspanin peptides affected HCMV infection (Figure 2B). These results suggest that proteins comprising the LELs of CD9, CD63, CD81 and CD151 have a weak inhibitory effect on HPV infection and no effect on HCMV infection.

### 2.3. Blocking Peptides of the Cytoplasmic C-Terminal Tail of Tetraspanins Inhibit HPV16 Infection.

**Figure 3:**
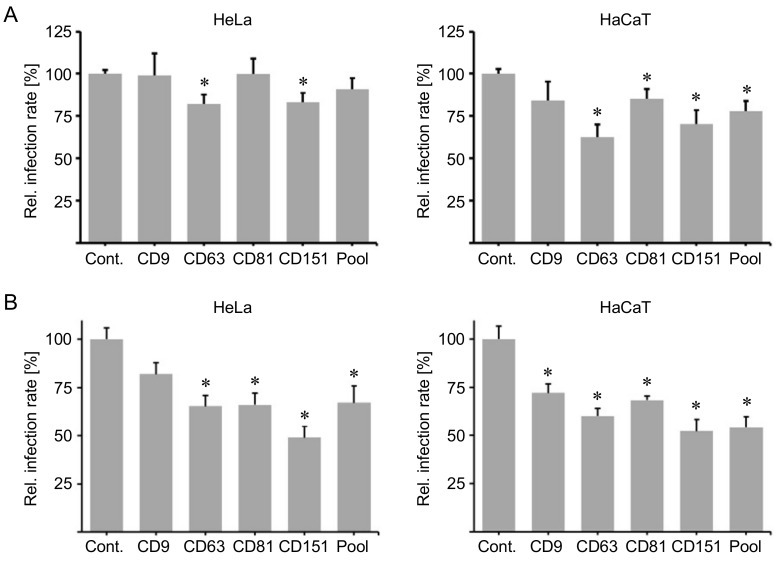
Blocking peptides of the cytoplasmic C-terminal tail of tetraspanins inhibit HPV16 infection. (**A**) HeLa and HaCaT cells were incubated with 2 µM cytopermeable control peptide (scrambled amino acid sequence of CD81; Contr.) or peptides comprising the cytoplasmic C-terminal tail of tetraspanin CD9, CD63, CD81, CD151, or a pool of the CD9, CD63, CD81 and CD151 peptides (Pool) using 0.5 µM of each peptide for 1 h. Next, the cells were infected with HPV16 PsVs and relative infection rate was assessed by measuring luciferase activity 24 h.p.i. (**B**) HeLa and HaCaT cells were treated as in A but the PULSin transfection reagent was present for 1 h to increase cytoplasmic delivery of the peptides. Next, the cells were infected with HPV16 PsVs and relative infection rate was assessed by measuring luciferase activity 24 h.p.i. The data is shown as mean ± standard error of the mean (SEM) and was normalized to the mean of control peptide treated cells (set to 100%). Asterisks indicate significant differences compared to control (*p* < 0.05).

The cytoplasmic C-terminal domain of tetraspanins is a functional region required for interaction with cytoplasmic partner proteins [15,16,17,18,19]. The C-terminus of CD63 facilitates complex formation between CD63 and its direct interaction partner syntenin-1 [16], which is required for post-endocytic trafficking of HPV [40]. Moreover, HPV infection involves the C-terminus of CD151 [28], which affects integrin functions and links CD151 to intracellular pathways [51,52,53,54]. Therefore, we used cytopermeable peptides comprising the C-terminal region sequence as inhibitors of tetraspanin functions. These peptides have been shown to block some tetraspanin-dependent functions having the same functional effect than that of siRNA silencing [18]. In that sense, HeLa and HaCaT cells were incubated with a control peptide comprising the scrambled combination of the ten amino acids of CD81 C-terminus (Cont.), as well as peptides containing the C-terminal sequence of tetraspanins CD9, CD63, CD81 and CD151, or a pool of these peptides and subsequently exposed to HPV16 PsVs. The C-terminal peptides of tetraspanins CD63 and CD151 significantly reduced HPV infection rates in HeLa cells 24 h.p.i. In HaCaT cells, the C-terminal peptides of tetraspanin CD63, CD81 and CD151 as well as the pool of all tested peptides significantly decreased HPV infection rates 24 h.p.i. (Figure 3A).

Although the recombinant peptides used are able to enter the cell [18], we applied the protein delivery reagent PULSin to test whether the inhibitory effect of C-terminal peptides could be enhanced by an increased cytoplasmic delivery of the peptides. HeLa and HaCaT cells were treated with peptides pre-incubated with PULSin for 1 h before exposure to HPV16 PsVs. Indeed, the addition of the PULSin reagent increased the inhibitory effect of HPV infection rates. In HeLa cells, C-terminal CD63, CD81 and CD151 peptides as well as the peptide pool significantly reduced HPV infection rates, whereas in HaCaT cells all tested C-terminal peptides induced a significant reduction of the HPV infection rate (Figure 3B).

In summary, the C-terminal peptides of tetraspanins CD63 and CD151 showed the strongest effects on infection rates.

**Figure 4:**
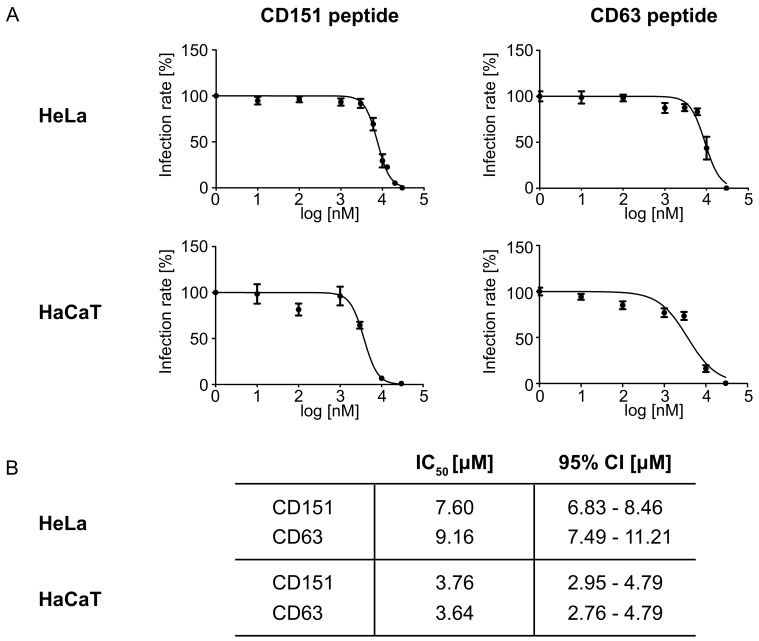
Dose-response curves (**A**) and IC_50_ with 95% CI values (**B**) of C-terminal peptides of CD63 and CD151 in HPV16 infection. Cells were incubated with C-terminal peptides at final concentrations ranging from 1 to 30,000 nM for 1 h. Afterwards, the cells were infected with HPV16 PsVs. Pseudovirus infection assays were performed 24 h after PsVs addition. The data is shown as mean ± SEM.

Next, the half-maximal inhibitory concentration (IC_50_) of the most effective peptides, CD63 and CD151, was determined. To exclude the cellular toxicity or other side effects of PULSin, the IC_50_ was performed without any enhancers of transfection. The IC_50_ categorizes inhibitors into three classes: potent (IC_50_ < 1 μM), moderate (1 μM < IC_50_ < 10 μM), or weak (IC_50_ > 10 μM) inhibitors [55,56,57]. The dose-response curves and calculated IC_50_ of the C-terminal CD63 and CD151 peptides in HPV infection as well as the assessed 95% confidence interval (95% CI) are shown in Figure 4.

### 2.4. Blocking Peptides of the Cytoplasmic C-Terminal Tail of Tetraspanins Inhibit Human Cytomegalovirus (HCMV) Infection.

**Figure 5:**
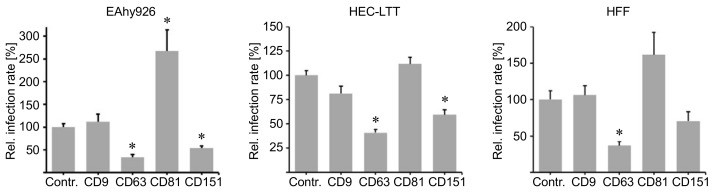
Blocking peptides of the cytoplasmic C-terminal tail of tetraspanins inhibit HCMV infection. EA.hy926, HEC-LTTs or HFF cells were incubated with 2 μM scrambled control peptide (Contr.) or peptides with the C-terminal tail of CD9, CD63, CD151 for 2 h at 37 °C. Afterwards, the cells were infected with the HCMV strain TB40/E and 24 h.p.i. fixed with 80% acetone. The fraction of infected cells, IE-Ag positive, was determined by immunofluorescence. The data is shown as mean ± SEM and was normalized to the mean of control peptide treated cells (set to 100%). Asterisks indicate significant differences compared to control (*p* < 0.05). Graphs represent data collected from two independent experiments.

To examine the inhibitory effect of the blocking peptides against the C-terminal tails of tetraspanins in a second viral infection model, EA.hy926, HEC-LTT (human endothelial cell-large T antigen and telomerase) and HFF (human foreskin fibroblast) cells were incubated with blocking peptides of the C-terminal tail of tetraspanins CD9, CD63, CD81, and CD151 or the control peptide and then exposed to the HCMV strain TB40/E. Peptides comprising the C-terminal CD9 and CD81 peptide had only minor effects on HCMV infection. However CD81 peptide significantly increased HCMV infection in EAhy926 cells, which might be a cell line specific effect. The peptide comprising the cytoplasmic C-terminal tail of CD151 significantly reduced HCMV infection rates in EA.hy926 and HEC-LTT cells, while the peptide of the cytoplasmic C-terminal tail of CD63 significantly decreased HCMV infection rates in all cell lines tested (Figure 5).

**Figure 6:**
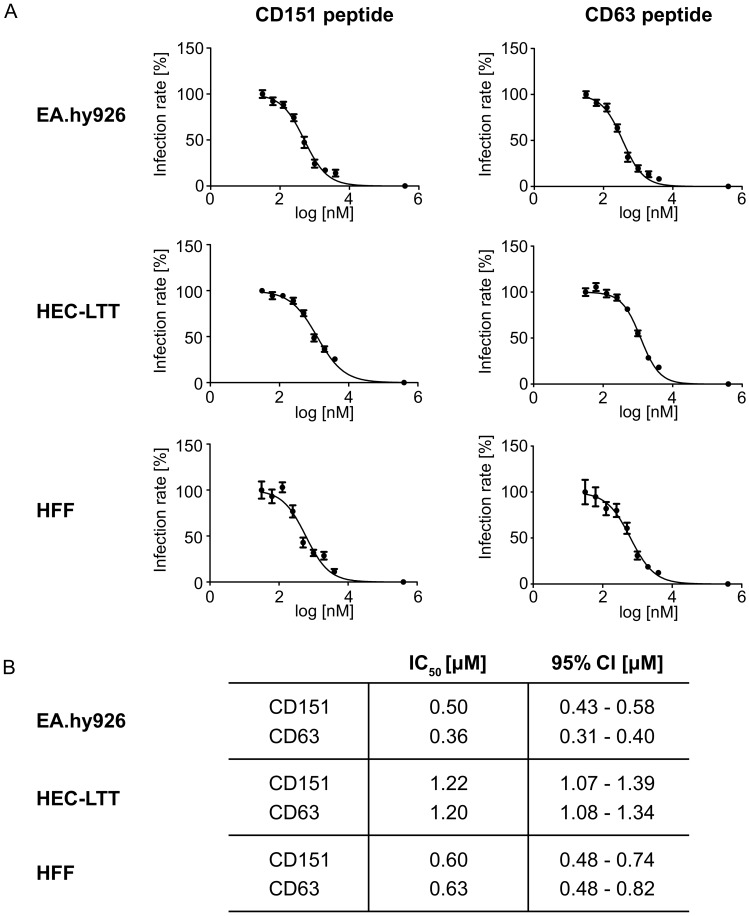
Dose-response curves (**A**) and IC_50_ with 95% CI values (**B**) of C-terminal peptides of CD63 and CD151 in HCMV infection. C-terminal peptides were used at final concentrations ranging from 30 to 4000 nM. The cells were incubated with peptide for 2 h, infected with TB40/E virus and incubated for 2 h at 37 °C. The fraction of IE-Ag positive cells was analyzed by immunofluorescence staining. The data is shown as mean ± SEM.

As found in HPV infection inhibition assays, peptides harboring the sequence of the C-terminus of CD63 and CD151 displayed the strongest effect. Based on these findings, the dose-response curves and IC_50_ of CD63 and CD151 in HCMV infection were determined as shown in Figure 6.

### 2.5. C-Terminal Peptides of CD63 and CD151 Inhibit HPV16 Entry.

**Figure 7:**
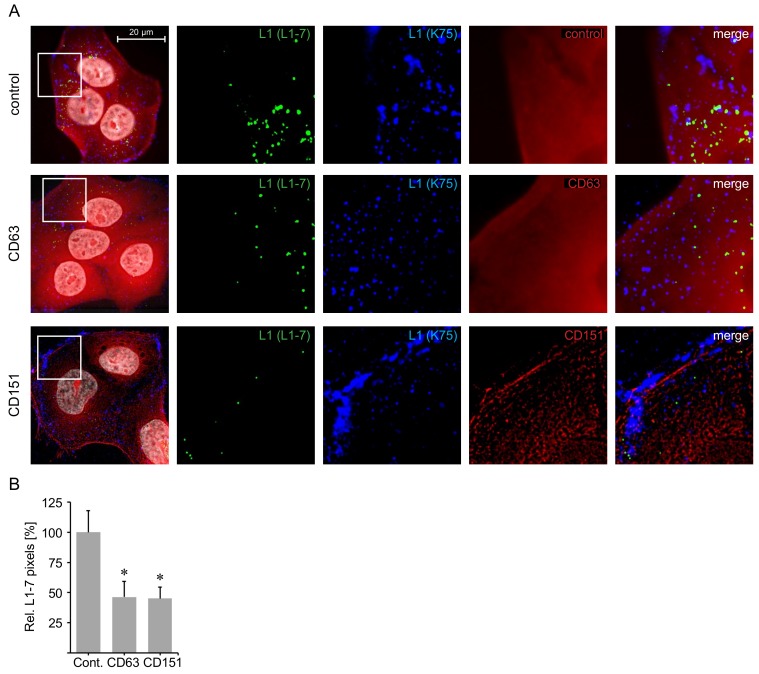
C-terminal peptides of CD63 and CD151 inhibit HPV16 entry. HaCaT cells were transfected with 2 µM of control peptide or peptides comprising the cytoplasmic C-terminal tail of tetraspanin CD63 or CD151, mixed with PULSin transfection reagent 1 h prior addition to the cells. Afterwards, the cells were incubated with HPV16 PsVs for 7 h and stained by immunofluorescence. (**A**) Representative pictures of immunofluorescence with HPV16 L1 monoclonal antibody L1-7 (green), polyclonal antibody K75 (blue), specified peptide (red) and Hoechst for DNA staining (white). L1-7 recognizes the HPV16 L1-7 epitope accessible after HPV internalization and capsid disassembly. K75 recognizes all bound and internalized HPV particles. (**B**) Quantification of relative L1-7 per DNA pixels. Data is shown as mean ± SEM and was normalized to the mean of control peptide (Cont.) transfected cells (set to 100%). Analysis was performed on 20 images per group using ImageJ software. Asterisks indicate significant differences compared to control (*p* < 0.05).

During HPV entry, the disassembly of the viral capsid is required for the delivery of the viral genome into the nucleus. CD63- and CD151-depleted cells show significantly decreased capsid disassembly [28,40]. To test whether C-terminal CD63 and CD151 peptides affect HPV capsid disassembly, we used the disassembly specific L1 antibody (L1–7) recognizing an epitope of the HPV major capsid protein L1 that is only accessible post internalization and capsid disassembly [27,58], as well as the polyclonal L1 antibody K75 detecting all bound and internalized viral particles [27,59,60] as a control for the total amount of viral particles. HaCaT cells were transfected with peptides, incubated with HPV16 PsVs for 7 h and analyzed via immunofluorescence. C-terminal CD63 peptide-treated cells showed the fluorescently labeled peptides throughout the cytoplasm, matching the cellular location of CD63 cytoplasmic interaction partner syntenin-1 [16,40]. In contrast, fluorescently labeled C-terminal CD151 peptides were observed in proximity to the plasma membrane, were L1 staining by polyclonal K75 antibody was mainly detected, suggesting that virus endocytosis was blocked by the CD151 peptides. Control peptide-transfected cells readily displayed L1–7 staining, as well as K75 staining. In C-terminal CD63- and CD151-peptide transfected cells L1–7 staining was significantly decreased compared to the control, whereas K75 staining was readily detectable (Figure 7). These results are in line with the indicated competitive inhibitor potency of C-terminal CD63 and CD151 peptides (Figure 3). Our results suggest that the inhibitory effect of C-terminal CD63 and CD151 peptides occurs prior to HPV capsid disassembly conforming to the role of CD63 and CD151 during HPV entry [28,40].

## 3. Discussion

A multitude of viruses require tetraspanin proteins for infection making tetraspanins compelling antiviral targets. In this study we compared the importance of tetraspanins CD9, CD63, CD81 and CD151 in infections by human papilloma- and cytomegalovirus and investigated whether peptides of functional tetraspanin domains could be used as inhibitors of HPV and HCMV infection.

Until now, the role of tetraspanins CD9 and CD81 in HPV infection remains poorly defined. Our experiments with siRNA-mediated knockdown of CD9 and CD81 in HeLa cells highlighted the importance of both tetraspanins in HPV16-mediated infection. CD9-targeting siRNAs reduced the HPV infection rate to an extent similar to the siRNA directed against CD151, whose crucial role in HPV infection was stated earlier [27,28,38]. A significant reduction of infection by nearly 90% after CD9 depletion makes this tetraspanin an interesting target of investigation for future HPV research. In comparison, CD81 depletion reduced HPV16 infection by approximately 30%. This was comparable to a previous study showing that the inhibitory effect of antibodies against CD81 was weaker than that of antibodies against CD63 and CD151 in HPV16 infection [27]. These results indicate that different tetraspanins have individual potencies in benefitting HPV infection. CD81 seems to play a minor role in HPV infection, whereas CD9 is likely to be a major player.

LELs are functional tetraspanin domains that have previously been shown to be involved in viral infection [14,29,61,62]. However, our data shows that soluble proteins comprising the LELs of CD9, CD63, CD81 and CD151 inhibit HPV infection to a significant but minor extent and pose no effect on HCMV infection. These findings are in strong contrast to the results reported for infection with human immunodeficiency virus (HIV), in which recombinant CD9, CD63, CD81 and CD151 LELs were identified as potent inhibitors [61]. Moreover, it was shown that recombinant CD81 LEL proteins are able to sufficiently bind Hepatitis C virus by interacting with its major virus envelope protein E2 [63], implying that LEL proteins are capable of competitively interacting with virions. Our results suggest that the LEL proteins do not capture HPV or HCMV virions nor interfere with other tetraspanin interactions involved in HPV and HCMV infection. Therefore, the antiviral potency of LEL proteins might be virus specific and requires further investigation.

More importantly, we show that cytopermeable peptides comprising the C-termini of CD63 and CD151 had a moderate to potent inhibitory effect on HPV16 and HCMV infections in different cell lines. Simultaneous addition of all tested tetraspanin peptides was unable to increase the effect of the individual peptides. Interestingly, CD9-targeting siRNAs caused a significant reduction of HPV (Figure 1) and HCMV [4] infections, whereas the C-terminal peptide had no or only a minor inhibitory effect. This discrepancy of the consequences of cellular depletion versus treatment of cells with CD9 C-terminal peptides might imply that CD9 affects HPV and HCMV infection via a function that does not involve C-terminal dependent cytoplasmic interactions. Therefore it is likely that CD9 benefits HPV and HCMV infection by organization of virus entry platforms (TEMs) [5] or the modulation of the activity or accessibility of interaction partners like proteases as it was proposed for entry of MERS viruses [64].

Overall, concentration curves of blocking peptides against the cytoplasmic C-terminal tail of tetraspanins CD63 and CD151 produced effective reduction in both viral infection systems and in all tested cell models, indicating that these peptides might be useful for development of HPV and HCMV inhibitors. This is in line with previous studies demonstrating that CD63 and CD151 are involved in HPV and HCMV infection and that the C-terminal domains of these tetraspanins are indispensable for their role in virus infection [4,27,28,38,40]. Moreover, the comparable effects of siRNA and C-termini treatment suggests that these two viruses take advantage of similar cellular pathways during infectious entry.

Mechanistically, we show that the CD63 and CD151 C-terminal peptides decreased the disassembly of HPV16 capsids which consequently inhibited infection. We observed differences in the cellular localization of the CD63 and CD151 peptides which might indicate the localization of the tetraspanin interaction partners. Direct interaction of CD63 C-terminus has been previously reported for the cytoplasmic adaptor proteins syntenin-1 [16] and AP subunits [17]. However, little is known about direct interactions facilitated by the CD151 C-terminus [12]. Moreover, distribution of the HPV16 capsid protein L1 indicates that C-terminal CD151 peptide is able to block virus endocytosis as the capsid protein was mainly detected in the cell periphery. On the other hand, cells treated with the CD63 peptide displayed evenly distributed capsid staining throughout the cytoplasm accompanied by reduced capsid disassembly suggesting inhibited intracellular trafficking and endosomal maturation. These findings are in line with the effect described for cellular depletion of these tetraspanins [28].

Our data suggests that the tetraspanin C-terminal peptides can specifically block the function of the corresponding tetraspanin domains, making them suitable reagents for investigating the role of tetraspanins in their diverse cellular functions. Furthermore, with assessed IC_50_ values lower than 1 µM for HCMV and lower than 10 µM for HPV, the C-terminal peptides of CD63 and CD151 can be classified as potent and moderate antiviral agents for HCMV and HPV infection, respectively, making them an interesting tool for entry prevention of other viruses.

## 4. Materials and Methods

### 4.1. Cell Lines and Pseudoviruses

The human cervical carcinoma cell line HeLa was purchased from the German Resource Centre for Biological Material (DSMZ, Braunschweig, Germany). The human nonvirally immortalized keratinocyte cell line HaCaT was purchased from Cell Lines Services (CLS, Eppelheim, Germany). Cells were grown in Dulbecco’s modified Eagle’s medium (DMEM + GlutaMAX, Thermo Fisher Scientific, Waltham, MA, USA) supplemented with 10% fetal calf serum (FCS), 1% minimum essential medium nonessential amino acids (MEM nonessential amino acids, Thermo Fisher Scientific, Waltham, MA, USA) and the antibiotic ciprofloxacin.

Human foreskin fibroblast (HFF), primary cells isolated from human tissue, were grown in MEM + GlutaMAX (Life Technologies, Darmstadt, Germany) supplemented with 5% FCS, basic fibroblast growth factor (0.5 ng/mL) and the antibiotic gentamicin (100 µg/mL).

EA.hy926 cells (ATCC CRL-2922), a hybrid cell line previously generated by fusion of human umbilical vein endothelial cells (HUVEC) and A-549 cells was purchased from LGC Standards (Wesel, Germany). Cells were grown in DMEM + GlutaMAX (Life Technologies), 10% FCS and antibiotic gentamicin [65].

Human endothelial cell-large T antigen and telomerase (HEC-LTT), a conditionally immortalized cell line generated from human umbilical vein endothelial cells (HUVEC) by introducing doxycycline-inducible gene expression cassettes for the simian virus 40 (SV40) large T antigen and the human telomerase catalytic subunit was kindly provided by Dagmar Wirth (Helmholtz Centre for Infection Research, Braunschweig). Cells were grown in EGM-BulletKit (CC-3124) (Lonza Sales Ltd., Basel, Switzerland): EBM (endothelial basal medium), bovine brain extract with heparin, human endothelial growth factor, hydrocortisone, ascorbic acid, GA-1000 (gentamicin, amphotericin B) and 2% FCS. For passaging the culture medium was supplemented with doxycycline (2 μg/mL) immediately before addition to the cells [65].

HPV16 pseudoviruses were prepared as described earlier [66]. HCMV strain TB40/E was originally isolated from a throat wash specimen obtained from a bone marrow transplant patient and subsequently propagated in HUVECs [67].

### 4.2. CD9, CD63, CD81 and CD151 siRNA Knockdown

CD9#1 (Hs_CD9_1), CD9#2 (Hs_CD9_7), CD81#1 (Hs_CD81_11), and CD81#2 (Hs_CD81_6) were purchased from Qiagen (Hilden, Germany). CD63#1 and CD151#1 were purchased from Sigma-Aldrich (St. Louis, MO, USA), and have been described previously [28,40]. Sequences are available upon request. Non-silencing control siRNA (AllStars Neg. Control siRNA, Qiagen) was used as control. HeLa cells were transfected with 30 nM siRNA using Lipofectamine RNAiMAX (Invitrogen, Carlsbad, CA, USA) for 48 h according to manufacturer’s instructions. Knockdown efficiency was quantified by Western blotting.

### 4.3. Antibodies

Mouse monoclonal CD9 and CD81 antibodies were purchased from Acris (Rockville, MD, USA) and Santa Cruz (Dallas, TX, USA), respectively. CD63 and CD151 antibodies were described earlier [28,40]. The HPV L1-specific antibodies mouse monoclonal antibody L1-7 and rabbit polyclonal antibody K75 were described previously [27,58,68]. Anti-IE-Ag (clone E13), mouse monoclonal antibody targeted against viral immediate early antigens 1 and 2 was purchased from Argene (Verniolle, France). Secondary, Cy3-conjugated goat polyclonal anti-mouse-Ig F(ab’)2 antibody was from Jackson ImmunoResearch (Baltimore, MD, USA).

### 4.4. LEL Proteins and C-Terminal Peptides

Recombinant fusion proteins mimicking the large extracellular loop of tetraspanins CD9, CD63, CD81 and CD151 as well as the glutathione *S*-transferase (GST) fusion partner were produced in *E. coli* and purified as previously described [50]. Peptides of the cytoplasmic C-terminal tail of tetraspanins CD9, CD63, and CD151 are tetramethylrhodamine (TAMRA) N-terminal–labeled peptides with the sequences RRRRRRRCCAIRRNREMV (CD9), RRRRRRRCCLVKSIRSGYEVM (CD63), RRRRRRRCLYRSLKLEHY (CD151) and were purchased from LifeTein (South Plainfield, NJ, USA) or DgPeptides (Hangzhou, Zhejiang, China). Peptides of the cytoplasmic C-terminal tail of CD81 as well as a CD81 scrambled negative control peptide have been described earlier [18,69].

### 4.5. HPV Inhibition Assay

HeLa and HaCaT cells were grown in 24- or 48-well plates and incubated with tetraspanin specific siRNAs, LELs or C-terminal peptides. Cells were infected with 100 luciferase vector positive HPV16 pseudoviruses per cell for 24 h. Luciferase expression control and HPV pseudovirus infection assays were performed as described previously [28,70]. Luciferase counts were normalized to lactate dehydrogenase (LDH) to take the cell viability into account.

To determine the effect of recombinant LEL proteins, HeLa and HaCaT cells were incubated with specified LELs in medium without supplements for 3 h before exposure to HPV16 PsVs. To determine the impact of C-terminal tetraspanin peptides two approaches were used:

(1) The cells were incubated with C-terminal tetraspanin peptide in medium without supplements for 1 h and subsequently infected with PsVs.

(2) The cells were transfected with a mixture of C-terminal tetraspanin peptide and a PULSin (Polyplus-transfection, Illkirch, France) preincubated for 1 h in medium without supplements before PsVs addition and were further cultured in medium with FCS. PULSin was used according to manufacturer’s instructions. 

### 4.6. HCMV Inhibition Assay

To determine the effect of proteins comprising LELs or C-terminal tails, the cells were incubated with specified reagents for 2 h before infection with HCMV strain TB40/E. The fraction of infected cells (IE-Ag positive cells) was detected with immunofluorescence staining applying primary antibody against immediate early antigen (IE-Ag) and Cy3-conjugated secondary antibody as described before [4]. Nuclei were counterstained with DAPI. A Zeiss Axio Observer D1 microscope (Zeiss, Jena, Germany) was used for visualization of fluorescence. Strain TB40/E was used at a multiplicity of infection (MOI) of 0.5 to 1.

### 4.7. IC_50_ Determination

To determine the IC_50_ (concentration necessary to achieve the half-maximal inhibition) for HPV, HeLa and HaCaT cells were incubated with C-terminal peptides at final concentrations ranging from 1 to 30,000 nM for 1 h in medium without supplements. Afterwards, the cells were infected with about 100 luciferase vector positive HPV16 pseudoviruses per cell. Pseudovirus infection assays were performed 24 h after PsVs addition as described above. Luciferase counts were normalized to lactate dehydrogenase (LDH) to take the cell viability into account. Analysis was performed using GraphPad Prism 5 Software, Inc. (San Diego, CA, USA) (www.graphpad.com). The data were normalized to the mean of the smallest tested concentration that was set to 100%.

To determine the IC_50_ for HCMV, C-terminal peptides were used at final concentrations ranging from 30 to 4000 nM. The cells were incubated with peptide for 2 h. Then, the cells were infected with TB40/E virus and incubated for 2 h at 37 °C. Afterwards, the medium containing the virus was exchanged by fresh medium supplemented with FCS. The fraction of infected cells, (IE-Ag positive cells) was then analyzed by immunofluorescence staining applying primary antibody for immediate early antigen (IE-Ag) and Cy3-conjugated secondary antibody. Analysis was performed using GraphPad Prism 5 Software, Inc. The data were normalized to the mean of the smallest tested concentration that was set to 100%.

### 4.8. Immunofluorescence

HeLa or HaCaT cells were grown on cover slips. After transfection and/or infection, cells were fixed and permeabilized in methanol. Afterwards, cells were incubated with primary antibody and species-specific Alexa-conjugated secondary antibodies (Invitrogen). Nuclear DNA staining was achieved by incubation with Hoechst 33,342 (Sigma-Aldrich). Cover slips were mounted on microscope slides using Dako Fluorescent Mounting Medium (Dako, Carpinteria, CA, USA). Images were acquired using Zeiss Axiovert 200 M microscope fitted with the Plan-Apochromat 100×/1.4 Oil objective and Axiovision deconvolution software (Carl Zeiss, Jena, Germany).

For determination of disassembled HPV16 PsVs (L1–7 epitope detection assay) HaCaT cells were grown on cover slips, transfected with C-terminal tetraspanin peptide for 1 h and infected with 100 HPV16 pseudovirus particles per cell for 7 h. Cells were fixed and permeabilized in methanol and stained with monoclonal antibody L1–7 as described in Spoden et al. 2008 [27]. Cover slips were mounted and images of 20 randomly selected cell islands per group were acquired as described above.

### 4.9. Statistics

If not stated otherwise, the experiments were repeated at least three times. Statistical analysis of all analyzable data points was assessed with two-tailed, unpaired *t*-tests using GraphPad Prism software and Microsoft Office Excel 2016. *p*-values smaller then 0.05 (*p* < 0.05) were considered as significant differences between groups.

## Abbreviations

CD	cluster of differentiation
CI	confidence interval
DAPI	4′,6-Diamidin-2-phenylindol
FCS	fetal calf serum
GFR	growth factor receptor
GST	glutathion-*S*-transferase
HCMV	human cytomegalovirus
HEC-LTT	human endothelial cell-large T antigen and telomerase
HFF	human foreskin fibroblast
h.p.i.	hours post infection
HPV	human papillomavirus
IC_50_	half-maximal inhibitory concentration
IE-Ag	immediate early antigen
LDH	lactate dehydrogenase
LEL	large extracellular loop
MOI	multiplicity of infection
PsV	pseudovirus
SEM	standard error of the mean
TEM	tetraspanin-enriched microdomain
